# On the selection of optimization parameters for an inverse treatment planning replacement of a forward planning technique for prostate cancer

**DOI:** 10.1120/jacmp.v3i3.2563

**Published:** 2002-06-01

**Authors:** Dimitre H. Hristov, Belal A. Moftah, Colette Charrois, William Parker, Luis Souhami, Ervin B. Podgorsak

**Affiliations:** ^1^ Department of Oncology King Faisal Specialist Hospital and Research Center P.O. Box 40047, Jeddah 21499, MBCJ64 Kingdom of Saudi Arabia; ^2^ Department of Medical Physics and Department of Radiation Oncology McGill University Health Centre 1650 Cedar Avenue Montreal Quebec Canada H3G 1A4

**Keywords:** prostate cancer, optimization parameters, inverse planning

## Abstract

The influence of organ volume sampling, lateral scatter inclusion, and the selection of objectives and constraints on the inverse treatment planning process with a commercial treatment planning system is investigated and suitable parameters are identified for an inverse treatment planning replacement of a clinical forward planning technique for prostate cancer. For the beam geometries of the forward technique, a variable set of parameters is used for the calculation of dose from pencil beams. An optimal set is identified after the evaluation of optimized plans that correspond to different sets of pencil beam parameters. This set along with a single, optimized set of objectives and constraints is used to perform inverse planning on ten randomly selected patients. The acceptability of the resulting plans is verified by comparisons to the clinical ones calculated with the forward techniques. For the particular commercial treatment planning system, the default values of the pencil beam parameters are found adequate for inverse treatment planning. For all ten patients, the optimized, single set of objectives and constraints results in plans with target coverage comparable to that of the forward plans. Furthermore inverse treatment planning reduces the overall mean rectal and bladder doses by 4.8% and 5.8% of the prescription dose respectively. The study indicates that (i) inverse treatment planning results depend implicitly on the sampling of the dose distribution, (ii) inverse treatment planning results depend on the method used by the dose calculation model to account for scatter, and (iii) for certain sites, a single set of optimization parameters can be used for all patient plans.

PACS number(s): 87.53.–j, 87.90.+y

## INTRODUCTION

From an obscure, highly specialized radiotherapeutic technique practiced in only a few specialized centers around the world, intensity modulated radiotherapy (IMRT) is developing into a mainstream radiotherapeutic technique available in most major radiotherapy centers. The IMRT technique relies on inverse treatment planning (ITP) and holds great promise for improving radiotherapy both through increased tumor control and decreased treatment morbidity. The current routine clinical use of IMRT is hindered by several difficulties, such as: complexity of equipment used for dose delivery, complexity of the ITP process, and quality assurance issues related with dose distribution calculation and dose delivery.

The clinical utility of an inverse treatment plan depends on several parameters, which are usually selected through an elaborate trial‐and‐error process that may involve several iterations of optimizations, volumetric dose calculations, and plan evaluations. Thus, even though ITP automatically designs the beam apertures and modifiers that are required for the delivery of a desired dose distribution, the process may be considerably more complex than the standard forward planning, whereby a dosimetrist designs the beam apertures and selects the physical beam modifiers.

In addition, the routine dosimetric quality assurance of the IMRT delivery is rather involved and time‐consuming because of: (i) the limitations of existing dosimetric verification systems; (ii) the possible need to perform quality assurance for every patient since there may be significant inter‐patient variability of intensity patterns and delivery sequences; and (iii) the uncertainty in the interpretation and in the clinical significance of differences between calculated and measured dose distributions.

The work presented in this paper focuses on the problem of parameter selection for inverse treatment planning. Such parameters account for organ volume sampling, lateral scatter inclusion as well as objectives and constraints used in the inverse treatment planning process. The density of dose‐calculation points required for analysis of treatment plans has been a subject of previous investigations and some controversy.^1^ ^–^ ^3^ However, these studies did not address the issue of point sampling for the purposes of the iterative inverse planning optimization of many beamlets. The following simplified example illustrates the importance of adequate dose sampling in inverse planning and its dependence on the number of beams involved.

Consider the case of a single target and a single beam port, covering generously the beam‐eye view of the target. Assume no attenuation. If a uniform dose is to be delivered to the target with no intensity modulation, a single sampling point anywhere in the target can be employed for optimization, since a uniform dose to that point guarantees a uniform dose to the target. However, if the same point is used for optimization of all beamlets across the beam port, the optimization will fail to deliver uniform dose to the target, since only few beamlets contributing dose to the particular sampling point will be activated. The rest will have zero weights, which will result in complete miss of the target.

Chen *et al*.^4^ have previously demonstrated that lateral scatter inclusion has to be accounted for in inverse planning if systematic target underdosage and unwanted injuries to critical organs immediately adjacent to the target are to be avoided. However, in their study no indication was given as to what extent (in terms of radial scatter distance) lateral scatter is to be included in the optimization.

Even though for certain sites inverse planning can certainly outperform forward planning,^5^ ^–^ ^8^ whether this is the case for a particular patient depends on the trial‐and‐error objective/constraint tuning,^9^ ^–^ ^12^ which is the most time consuming step in the design of clinically acceptable IMRT plans. This step is further complicated by the fact that IMRT plans often tend to depart from established clinical norms with respect to the target dose heterogeneity (15%‐20%).^7^ An appealing approach to shorten the trial‐and‐error process is to identify a single, common set of optimization criteria and parameters that can be applied at least as a first approximation to all patients undergoing a particular type of treatment, especially for sites that exhibit common target/organ configurations, such as prostate.

In this paper we present an approach that identifies adequate ITP organ sampling and scatter inclusion parameters. Even though these parameters are determined for a particular commonly used treatment planning system, their magnitude should indicate reasonable values of the same parameters for other planning systems. Furthermore, without necessarily aiming to produce “the best” IMRT plan, for the particular optimization algorithm, we show that ITP with a single set of objectives and constraints is possible for a particular treatment technique for prostate cancer. In order to illustrate this possibility, for ten patients, we compare inverse treatment plans generated with a single set of optimization parameters to their forward counterparts. Our goal is not to illustrate the superiority of inverse to forward planning, but to demonstrate that the inverse plans generated with a single set of parameters are clinically acceptable since they compare favorably to forward, clinically accepted plans. The methods presented in this paper can be extended to other inverse treatment planning systems and other anatomical sites.

## MATERIALS AND METHODS

### A. Treatment planning system

A commercial planning system (CadPlan 6.1.5, Dosetek, Varian, Palo Alto, CA) was used for the study. This system employs a pencil beam convolution model for volumetric calculation of the dose distribution on a regular 3D grid. Within this model, the absorbed dose *D(x,y,z;F)*, normalized to the absorbed dose delivered by a $10\times 10{\text{cm}}^{2}$ open field at reference depth $z_{\text{ref}}$ is given by

(1) $$D(x,y,z;F)={\left(\frac{f+z_{ref}}{f+z}\right)}^{2}\int \int K_{s}(x-x^{\prime },y-y^{\prime },z)P_{\mathrm{int}}(x^{\prime },y^{\prime },z)F(x^{\prime },y^{\prime })dx^{\prime }dy^{\prime },$$

where *f* is the source‐surface distance, $K_{s}(x,y,z)=K_{s}(r,z)$ is a radially symmetric dose spread kernel (DSK) which accounts for scatter at depth *z* in phantom, F=F(x,y) is the beam intensity distribution, and $P_{\mathrm{int}}(x,y,z)$ is the open (“flat”) beam fluence of primary photons at depth *z*. The function $P_{\mathrm{int}}(x,y,z)$ accounts for the nonflatness of the beam and for variations of the open beam fluence with depth *z* in phantom.

In order to determine the optimal intensity distribution F=F(x,y), the Helios ITP option of the CadPlan treatment planning system employs a gradient algorithm, which minimizes a square dose objective under “soft” dose and dose‐volume constraints.^13^ The dose distributions within each anatomical structure of interest are precalculated for randomly distributed sampling points. The user determines the number of sampling points within each structure. The dose $d_{p}$ deposited to a sampling point *P* is given as a vector equation by

(2) $$d_{p}=a_{p}.F,\;\;\;\;\text{with}\;\;a_{p}=\{a_{p}^{i,j}\},\;\;\;\text{and}\;\;\;\;F=\{F_{i,j}\}=F(x_{i},y_{j}),$$

where, after discretization, $F_{i,j}$ is the intensity of the beam at location $\left(x_{i},y_{j}\right)$, within the beam aperture and $a_{p}^{i,j}$, is the precalculated dose deposited to point *P* by a beam with intensity distribution $F^{i,j}=\delta_{i,j}$. The precalculated set $a_{p}^{ij}$ for fixed indices (*i,j*) is referred to as a pencil beam (*i j*) and $F_{i,j}$ is referred to as pencil beam weight.

In addition to the volume sampling rate, by supplying a single cut‐off parameter, the treatment planning system allows the user to modify the inclusion of lateral scatter in the pencil beam calculation. The cut‐off parameter limits the radial extent r of the dose‐spread kernel $K_{s}(r,z)$, which is convolved with the primary fluence in the dose calculation process.

**Figure 1:**
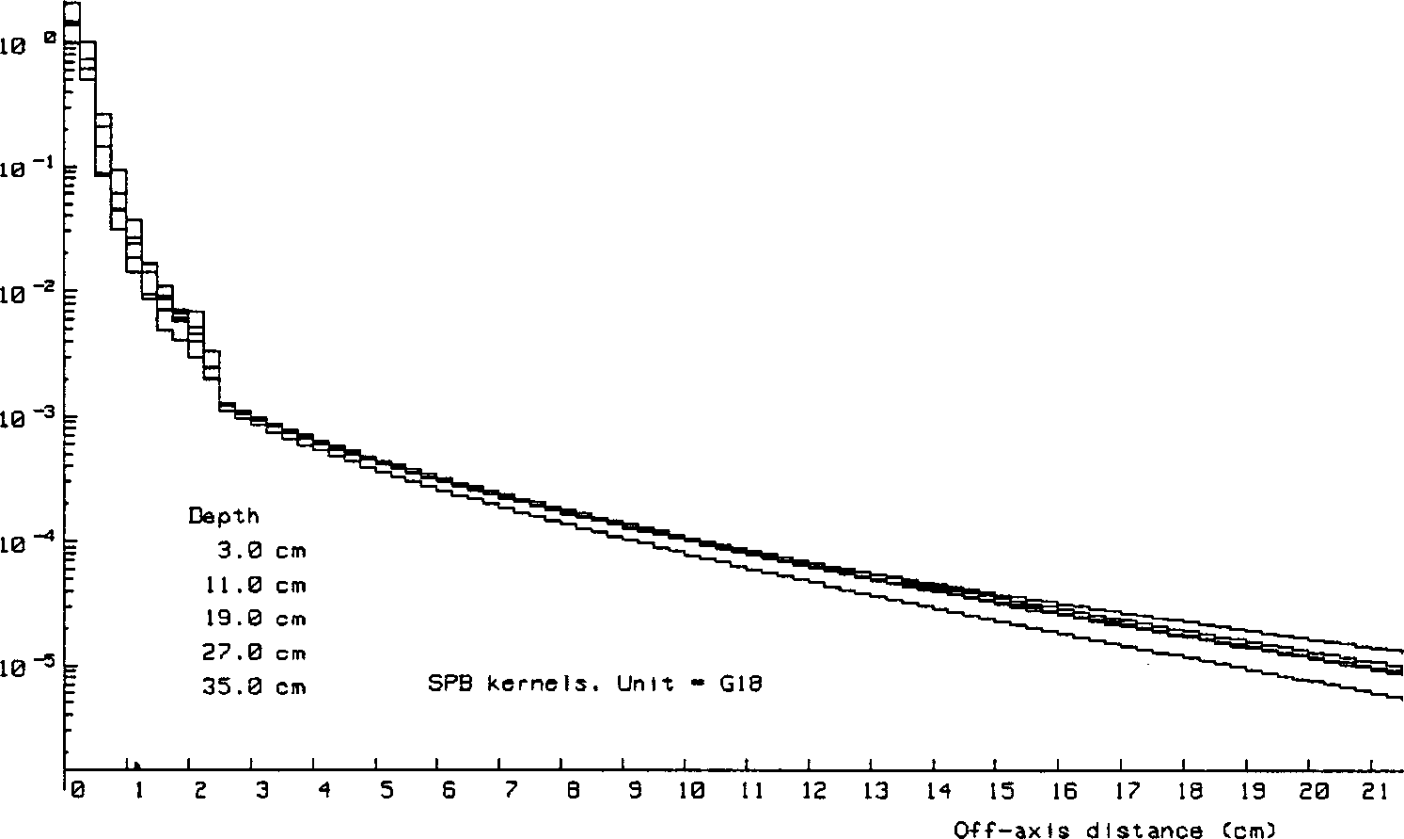
Radial dependence of the magnitude of the dose‐spread kernel for an 18 MV unit at various depths in water. The values are normalized to unity on the central axis of the pencil beam.

Figure 1 demonstrates the radial dependence of the dose‐spread kernel $K_{s}(r,z)$ at several depths for the particular 18 MV unit used for the planning of the cases in this study. The introduction of a cut‐off on the radial extent *r* of the dose spread kernel $K_{s}$ results in nullifying the dose spread kernel $K_{s}(r,z)$ for radial distances larger than the cut‐off value.

Once the pencil beam parameters are specified, the user defines treatment objectives and constraints. The objectives are formulated in terms of the admissible minimum and maximum target dose levels (in Gy) and their priorities (ranging from 0 to 100). The constraints with respect to critical structures fall into two categories: (i) dose constraints that stipulate the maximum admissible dose to the structure (in Gy) along with a priority (ranging from 0 to 100) and (ii) dose‐volume constraints that stipulate the relative volume of the structure (in%) that can be exposed above a certain dose level (in Gy) along with a priority (ranging from 0 to 100).

Thus, for the case of dose constraints only, the objective (cost) function $C_{obj}(F)$ to be minimized reads:

(3) $$C_{obj}(F)=\sum_{p=1}^{N}\xi_{p}w_{p}{\left(a_{p}\cdot F-D_{p}\right)}^{2},$$

where $w_{p}$ is the priority of a constraint or an objective, $D_{p}$ is a dose level of a constraint or an objective, *N* is the total number of dose sampling points, and the flag $\xi_{p}$ has the value of 1 if a constraint/objective is violated and 0 if it is not. For the case of dose‐volume constraints, the objective function is identical to that given by Eq. (3) but the summation is carried only over these sampling points which, when sorted in ascending order of dose received, cause the dose‐volume limit to be exceeded.

Having specified all parameters, the user starts the optimization process with the precalculation of the pencil beams, followed by the iterative adjustment of the pencil beam weights in the process of objective‐function minimization. At its conclusion, inverse planning provides the modulated intensities of the beams as fluence matrices or maps. These optimized fluence maps are then used as inputs to a leaf motion calculator, which determines a dynamic leaf sequence for each field. These leaf sequences are then used for the calculation of deliverable fluence maps and a complete volumetric calculation of the dose distribution on a regular 3D grid [see Eq. (1)] with no cut‐off imposed on the DSK (full inclusion of scatter, within a radial extent *r* of about 22 cm).

### B. Treatment planning techniques

Ten patients, who had recently undergone radiation treatments for the carcinoma of the prostate in our center, were randomly selected for the study. The treatment technique consisted of initial delivery of 45 Gy to planning target volume I (PTV1) in 25 daily fractions, followed by a boost of 25.2 Gy to planning target volume II (PTV2) in 14 daily fractions. PTV1 encompassed the prostate (GTV) with a uniform 1.5 cm margin, and PTV2 encompassed the GTV with a uniform 0.5 cm margin.

**Figure 2:**
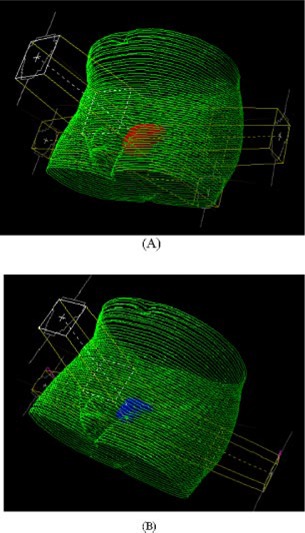
(Color) Beam arrangements for a typical patient. (a) Plan 1. (b) Plan II. For clarity only PTV 1 (a) and PTV 2 (b) are shown.

With the patient in supine position, a four‐field box technique (Plan I) with MLC‐shaped conformal beams and wedges (if necessary) was used for the initial delivery. In Plan II, a three‐field technique with one anterior (gantry angle 0°) and two posterior lateral fields (gantry angles 260° and 100°) was employed for the boost treatment of the patient in supine position. The beam energy for both plans was 18 MV. Both Plan I and Plan II were optimized manually by continuous adjustments of the MLC apertures and of the beam modifiers (physical wedges) until the PTV for the corresponding plan was encompassed as tightly as possible by an isodose surface at 95% of the plan prescription dose. This optimization criterion corresponds to an attempt to spare the bladder and the rectum as much as possible without violating the ICRU recommendation of target dose uniformity, given as –5% to +7% of the prescription dose.^14^ A typical example of a forward treatment plan is shown in Fig. 2, where Fig. 2(a) represents the beam setup for the treatment of PTV1 (Plan I) and Fig. 2(b) represents the beam setup for the treatment of PTV2 (Plan II).

The ten randomly selected patients were replanned with ITP, which employed the treatment unit, beam energy, and beam geometries used by the clinical forward planning techniques. The treatment unit is equipped with a 52‐leaf MLC with a leaf size of 10 mm at the treatment unit isocenter. The dimensions of the optimized fluence are 26×256 with a pixel size of 10×2.5 mm at the isocentre. Dynamic delivery of 250 segments per field was specified for the leaf motion calculator.

### C. Evaluation of optimization parameters

First, the sensitivity of the ITP with respect to the pencil‐beam parameters, the volume‐sampling rate of the anatomical structures, and the radial cut‐off of the DSK was evaluated. The volume sampling rates were varied to cover the range from $5\;\text{pts}/{\text{cm}}^{3}$ to $100\;\text{pts}/{\text{cm}}^{3}$. The radial cut‐off of the DSK was varied to cover the range from 0.16 to 2 cm. For one of the ten patients we performed ITP for several values of the above parameters while keeping the same objectives and constraints. The resulting intensities were fed into the leaf motion calculator, and the calculated deliverable fluence maps were then used for the complete volumetric calculation of dose distributions on a regular grid with a resolution of 2.5 mm.

These dose distributions were evaluated in terms of the dose statistics and the cumulative dose‐volume histograms (DVHs) of the various structures in order to determine adequate values for the volume‐sampling rate and the radial cut‐off of the DSK. The dose‐volume histograms were computed from the volumetric dose distribution with the same dose grid for all forward and inverse plans.

Having identified these values for a single patient, we then performed ITP for both Plan I and Plan II for various sets of objectives and constraints. The goal was to achieve target coverage comparable to that attained by the forward planning (FP) technique. The selected optimal dose/priority parameters are listed in Table I. The same set of optimization parameters was used for the inverse planning of Plans I and II. These parameters were subsequently used as an input for the ITP of all ten patients. Both the conventional and the inverse plans were normalized to deliver the prescription doses to the ICRU 50^14^ isocenter prescription point.

**Table I:** Dose and priority parameters used for inverse treatment planning.

Organ	Minimum dose (%)/Priority	Maximum dose (%)/Priority
PTV1, PTV2	100/100	100/100
Rectum		
Bladder		77/50

## RESULTS AND DISCUSSION

**Figure 3:**
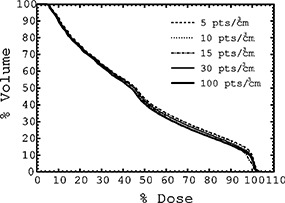
DVHs of the rectal volume for boost plans (Plan II) obtained with different pencil‐beam volume‐sampling rates at the optimization stage.

Figure 3 illustrates the sensitivity of the rectal DVHs with respect to the pencil‐beam volume‐sampling rates used for inverse treatment planning of the boost treatment for a typical patient. The radial DSK cut‐off was set at 2.0 cm. With an increasing volume‐sampling rate, the DVHs approach the DVH for a volume‐sampling rate of $100\;\text{points}/{\text{cm}}^{3}$. A volume‐sampling rate of $100\;\text{points}/{\text{cm}}^{3}$ should be adequate for the optimization, since for this sampling rate, the distance between the sampling points is smaller than the spacing of 2.5 mm between the nodes of the grid used for the final volumetric dose calculation. For the bladder and PTV2, similar behavior of the DVHs was observed.

Since the dose calculation time at each iteration is directly proportional to the point density within the anatomical structures of interest, the choice of an adequate volume‐sampling rate is a compromise between the conflicting requirements for a minimal optimization time and an accurate representation of the optimization results for a given set of objectives and constraints.

In order to speed up the optimization process, one can select a volume‐sampling rate as low as $5\;\text{points}/{\text{cm}}^{3}$ if one judges the differences between the DVHs for $5\;\text{points}/{\text{cm}}^{3}$ and for $100\;\text{points}/{\text{cm}}^{3}$ to be clinically insignificant. However, given the current clinical knowledge, such a judgment is bound to be rather subjective. Instead, based on the DVH differences revealed in Fig. 3, we consider the default manufacturer's value of $30\;\text{points}/{\text{cm}}^{3}$ to be adequate for inverse treatment planning since, for any dose point, the volume predicted by the DVH for $30\;\text{points}/{\text{cm}}^{3}$ differs by no more than 2% from the volume predicted by the DVH for $100\;\text{points}/{\text{cm}}^{3}$. The 2% criterion is quite stringent, given that the volume determination accuracy in radiation therapy planning is most probably in the 5%–10% range, due to both the interobserver variability^15^ and the finite‐slice thickness employed in CT/MR image acquisition.^16^

It is clear from the simple example presented in the Introduction that volume‐sampling rate is correlated to the effective cross‐section size of the pencil beams employed for the optimization. For the purposes of tumor control probability estimation Niemierko and Goitein^1^ suggest that the volume sampling rate should correspond to an average interpoint distance equal to a fraction (1/3) of the beam penumbra. However, in analogy with the Nyquist condition as applied to CT reconstruction, for the case of inverse planning one may require that the average interpoint distance be such that at least two points are contained within the cross‐section of a pencil beam. Such a requirement will also assure that a pencil beam “senses” both a target and a critical organ in immediate vicinity to each other. However, the interpretation of the pencil‐beam cross‐section size is not unequivocal, since it can be taken at various dose levels relative to the dose maximum. Considering only the geometric pencil‐beam size (0.$25\;25\;\times \;25\;\text{cm},8\;\text{pts}/{\text{cm}}^{2})$, one estimates a volume‐sampling rate of about $23\;\text{pts}/{\text{cm}}^{3}$, in general agreement with the DVH findings.

**Figure 4:**
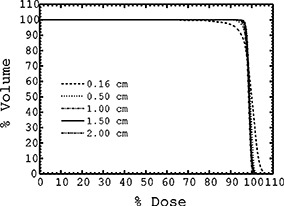
DVHs of the target volume (PTV1) for Plan I obtained with different DSK radial cut‐offs at the optimization stage. Plan I delivers 45 Gy to the 100% (isocenter).

Figure 4 demonstrates the sensitivity of the DVHs for the initial target volume (PTV1) with respect to the DSK radial cut‐off used in the calculation of the pencil beams for the optimization process. The inclusion of lateral scatter beyond 1 cm of the pencil‐beam ray line results in minor differences between the corresponding DVHs. Similar observations were made for the bladder and the rectal DVHs. Therefore, the inverse treatment planning time can be reduced by decreasing the DSK radial cut‐off down to 1 cm without compromising the accuracy of the calculated beam intensities. Such a choice is also appealing given that the total numbers of points that receive dose contribution from a particular pencil beam is proportional to the square of the radial cut‐off parameter. (The number of points is equal to the product of the point density and the volume, whereby a cylinder with a radius equal to the cut‐off parameter can approximate the “dose‐activated” volume around a pencil beam).

**Figure 5:**
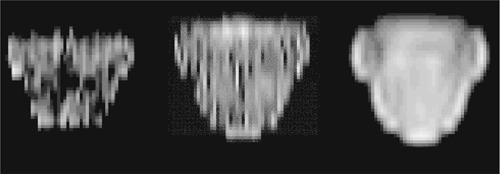
An example of the variation of the deliverable fluence patterns resulting from different values of the radial cut‐off parameter. The fluence patterns are for the anterior field employed in Plan II. The values of the cut‐off parameter from left to right are: 0.16, 0.5, and 1.5 cm.

However, if the 2% criterion used in the analysis of the pencil‐beam volume‐sampling rates is applied to identify clinically identical DVHs in Fig. 4, only the DVHs for a cut‐off of 1.5 cm and for a cut‐off of 2.0 cm are identical. Thus, we consider the default system DSK radial cut‐off value of 1.5 cm to be adequate for inverse planning with 18 MV beams. However, we would like to point out that, for other beam energies, the DSK radial cut‐off value may change since the dose‐spread kernel (Fig. 1) varies with the beam energy. For instance, one might expect to be able to decrease the DSK cut‐off value when 6 MV beams are employed for inverse planning. In this scenario, contrary to the case of 18 MV beams, laterally scattered electrons are not energetic enough to deposit dose at relatively large radial distances and therefore the DSK radial cut‐off value may be amenable to reduction without compromising the quality of the optimization results. Figure 5 illustrates further that inadequate inclusion of scatter in the dose calculation model used in inverse planning results in irregular, somewhat shrunk intensity patterns that underdose the target volume, particularly at the edges of the target as seen in the beam‐eye view.

**Figure 6:**
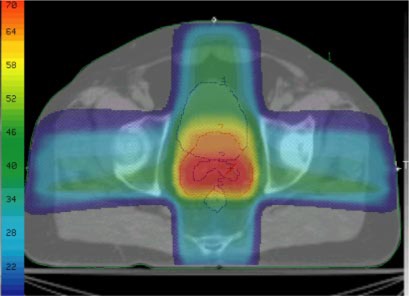
(Color) Color wash display of the dose distribution for the composite plan in an axial plane at the level of the seminal vesicles. PTV 2 (contour #2), PTV 1 (#7), rectum (#5), and bladder (#4) are shown. The color scale is in Gy

**Figure 7:**
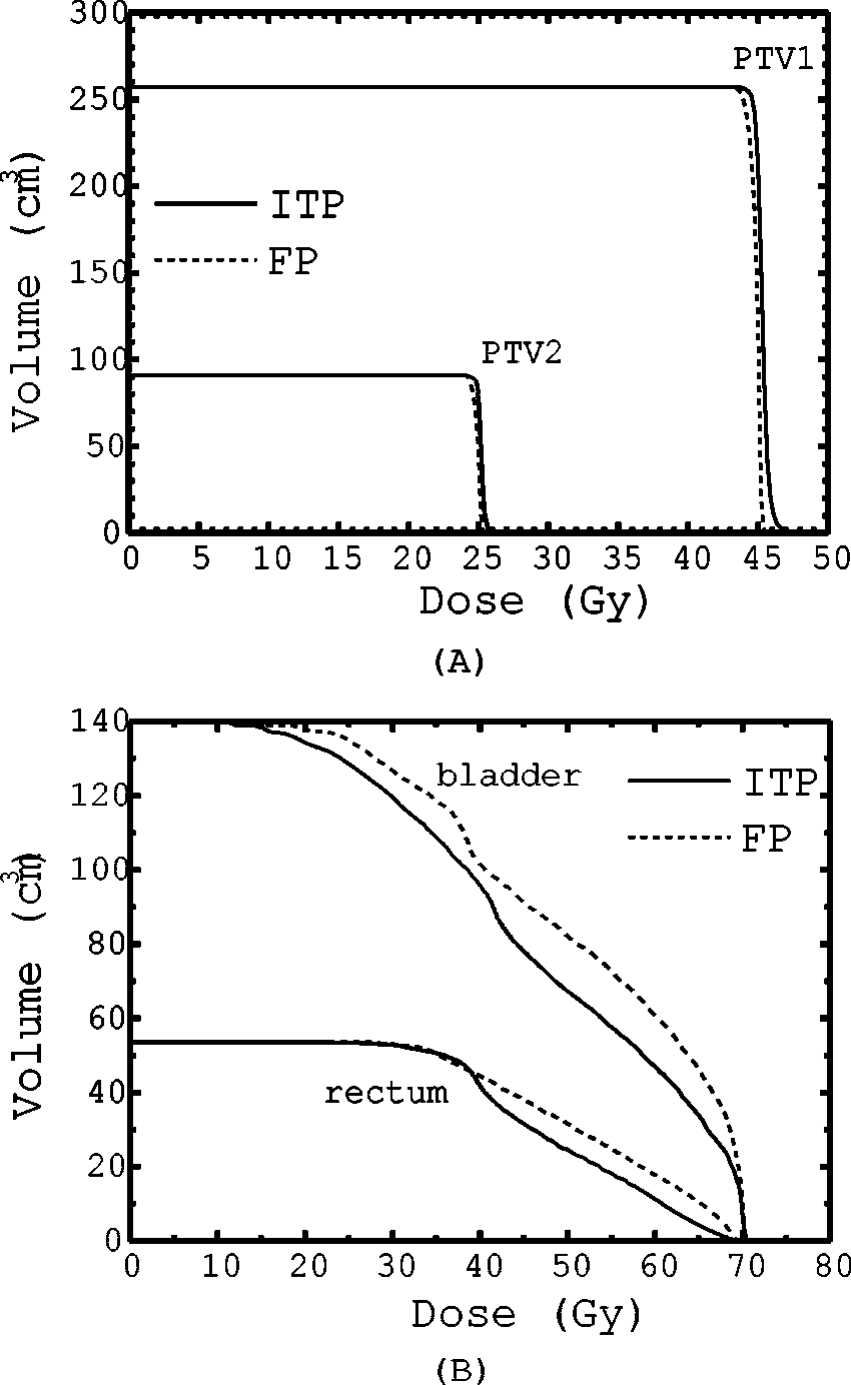
(a) DVHs of the target volumes PTV1 and PTV2 for plans created by the forward planning technique (FP) and the inverse treatment planning technique (ITP) for one of the ten patients in the study. (b) DVHs of bladder and rectal volumes for the cumulative plans (Plan I and Plan II) created by the forward planning technique (FP) and the inverse treatment planning technique (ITP) for the same patient.

For the ten patients used in this study, using the volume sampling rate of $30\;\text{points}/{\text{cm}}^{3}$, the radial DSK cut‐off of 1.5 cm, and the single set of dose/priority parameters listed in Table I, we generated inverse plans and evaluated their clinical acceptability by comparing them to their forward planning counterparts. A composite inverse plan (sum of inversely planned phase I and II) is shown in Fig. 6. Examples of DVHs obtained by the clinical forward planning (FP) and its ITP counterpart are shown in Figs. 7 for a typical patient; with the ITP curves shown as solid lines and the FP curves shown as dashed ones. For this patient, at the expense of increased target dose inhomogeneity, the ITP technique provides equal or slightly better coverage of the target volumes than does the FP [Fig. 7(a)] with some reduction of the doses delivered to the bladder and the rectum [Fig. 7(b)].

**Figure 8:**
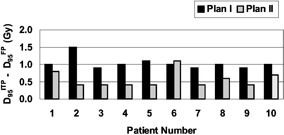
Plot of the differences in the doses $\left(D_{95}\right)$ delivered to 95% of the target volumes PTV1 and PTV2 by the ITP and by the FP

Similar observations hold true for the majority of the patients in this study. Figure 8 demonstrates that, for both Plan I and Plan II, and for all ten patients, the ITP based on a single set of objectives and constraints provided comparable or better coverage (as measured by the dose delivered to 95% of the target volumes) than did the FP. Concerning the target dose inhomogeneity, the “hot spots” in the planning target volumes (PTVs) were evaluated. For all ten patients, the “hot spots” were judged clinically insignificant, since their magnitudes were smaller than the ICRU 5014 limit of +7% with respect to the prescription dose.

The inverse plans based on a single set of objectives and constraints were also evaluated for consistency and clinical acceptability with respect to the bladder and rectal sparing. DVH and isodose analyses confirmed that adequate, clinically acceptable sparing of the bladder and the rectum was achieved for all patients.

**Figure 9:**
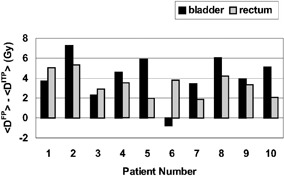
Plot of the differences in the mean bladder and rectal doses ((D)) delivered by the FP and by the ITP cumulative (Plan I and II) plans.

Figure 9 summarizes these findings by demonstrating that, with the exception of one patient (#6), the ITP resulted in plans with mean doses to the rectum and the bladder lower than the mean doses delivered to these organs by the FP technique. On average, the ITP reduced the mean dose to the rectum by 3.4 Gy (~4.8% of the prescription dose) and to the bladder by 4.1 Gy (~5.8% of the prescription dose).

**Figure 10:**
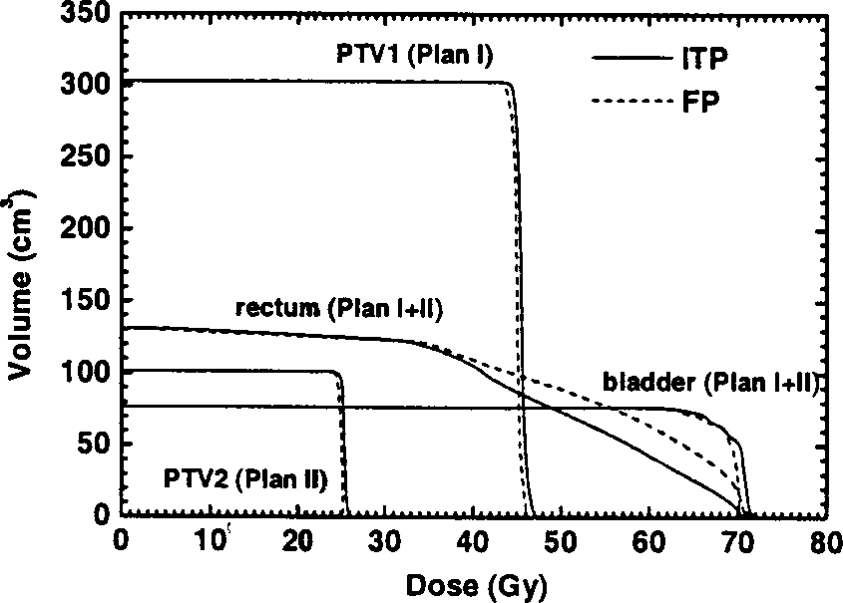
DVHs for patient #6 for whom the ITP underperformed the FP in terms of bladder sparing.

**Figure 11:**
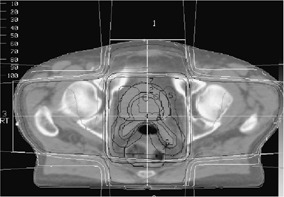
An axial CT image of patient #6, illustrating the significant overlap between the targets (PTV1 is contour #7, and PTV2 is contour #2) and the bladder (contour #5).

We further investigated the forward and inverse plans for patient #6 in order to correlate the only failure of the single‐parameter ITP to outperform the forward planning in terms of bladder sparing with particular features of the anatomy and the treatment geometry. Figure 10 compares inverse treatment planning and forward planning DVHs for the two target volumes (PTV1 and PTV2), the bladder and the rectum and demonstrates that the difference between the ITP and FP bladder DVHs is rather small and probably clinically insignificant. More importantly, almost the entire bladder volume for this patient is raised to a dose closer to the prescription (Fig 10). A review of the target and the critical organ volumes revealed that for this particular patient, PTV1 was almost entirely covering the bladder volume, as illustrated in the single axial image presented in Fig. 11. Thus, ITP had to compromise significantly the bladder volume, since the target had a larger priority (Table I) in the optimization process.

It is worth noting that in addition to the dosimetric advantages offered by the plans optimized by ITP, the total time required for ITP with a single set of objectives/constraints and the default cut‐off and sampling parameters is about 15 min, in comparison to approximately 30–40 min required by experienced dosimetrists or by clinical physicists to perform the forward clinical planning. However, it also must be pointed out that, for other inverse treatment planning systems, the ITP process can be more or less time‐consuming depending on the efficiency of the optimization engine.

## CONCLUSIONS

We have presented an approach to identify optimal values of the parameters that determine the organ sampling and the scatter inclusion to be used in the process of inverse treatment planning. While a particular treatment planning system was used to investigate the sensitivity of the inverse treatment planning process with respect to these parameters, the results presented here should indicate reasonable values for analogous parameters used in other treatment planning systems. As exemplified by the treatment of scatter in this paper, shortcuts taken by the vendors may result in consistently suboptimal treatment plans irrespective of the actual dose or dose‐volume constraints employed. Thus, while certain commercial systems may not provide the user with the capability of modifying the parameters related to organ sampling and scatter inclusion, their vendors should at least provide the default values.

By the example of a particular treatment planning technique, we have further demonstrated that routine inverse treatment planning based on a single set of objectives and constraints is a possibility for certain treatment sites and techniques. This finding suggests that after a proper parameter selection (organ sampling, scatter inclusion, objective, and constraints), inverse planning with a fixed set of parameters can serve as a useful replacement of some routine forward planning techniques.

## ACKNOWLEDGMENT

This work was partly supported by MRC Grant No. MOP 36470.
